# Shaping the future of cardiac interventions and cardiac surgeries: The impact of virtual reality and artificial intelligence

**DOI:** 10.21542/gcsp.2025.37

**Published:** 2025-08-30

**Authors:** Antoine Fakhry AbdelMassih, Abdullah Faris Nasser, Gawahir AbdelRahman, Lama Ebrahim Mkarem, Mariam Mousa AbuShashieh, Rahaf AbuGhosh, Emad Gamaleldin Nasr

**Affiliations:** 1 Pediatric Cardiology Unit, Pediatrics’ Department, Faculty of Medicine, Cairo University, Cairo, Egypt; 2 Pediatric Cardiology Division, Cardiac Sciences Department, SKMC, Abu Dhabi, United Arab Emirates; 3 Pediatric Residency Program, Pediatrics’ Department, SKMC, Abu Dhabi, United Arab Emirates; 4 Bachelor of Jordan University of Science and Technology, Amman, Jordan; 5 Aswan Heart Center, Aswan, Egypt

## Abstract

**Background:** Virtual reality (VR) and artificial intelligence (AI) have had a profound impact on transforming cardiac interventions by enhancing procedure planning, execution, and medical education. Virtual reality enables healthcare professionals to refine their skills by practicing procedures in a simulated environment while also improving patient understanding of their conditions. Artificial intelligence enhances diagnosis and treatment planning by analyzing patient data, detecting patterns, and improving both accuracy and personalized care. The aim of this review was to analyze the anatomical scopes of both technologies in the context of cardiac interventions as well as the radiologic modalities involved in image reconstruction in virtual reality.

**Methodology:** A literature search using the keywords “reviews”, “artificial intelligence”, ”virtual reality”, and “cardiac interventions” was conducted across PubMed, Scopus, and Google Scholar. The search was limited to English-language systematic reviews; narrative reviews, individual research articles, editorials, and opinion papers were excluded.

**Results:** An analysis of three reviews encompassing 71 studies revealed the applications of virtual reality (VR) and artificial intelligence (AI) in cardiac surgery. VR training was most frequently applied to mitral valve repair, while VR planning was most common for conotruncal anomalies. AI-driven decision support was most prevalent in heart transplantation.

**Conclusion:** This article highlights the established roles of virtual reality and artificial intelligence in cardiac care, encompassing surgical training, procedural planning, risk assessment, and outcome prediction. However, current VR training methods often rely on time-consuming and expensive imaging techniques like CMR and CT angiography. Within cardiology, AI-driven decision-making is most prominent in heart transplantation.

## Background

Virtual reality (VR) and artificial intelligence (AI) are increasingly important in the field of cardiac interventions^1^. These technologies can potentially enhance medical training, improve surgical planning, and enable more precise procedures. By leveraging virtual reality, medical professionals can simulate complex surgical scenarios and practice difficult procedures in a safe and controlled environment^2^. Artificial intelligence, on the other hand, can assist in interpreting medical images, predicting patient outcomes, and optimizing treatment strategies. As these technologies continue to advance, they are expected to play a significant role in advancing the field of cardiac interventions and improving patient care^3^.

Virtual reality technology has the potential to effectively address interpersonal gaps in surgical skills by providing a realistic and immersive training environment. By simulating surgical procedures and scenarios, VR can help bridge the gap in skills and experience among surgeons, ultimately leading to improved patient outcomes^4^.

Moreover, artificial intelligence plays a crucial role in predicting patients’ outcomes through the analysis of vast amounts of medical data. By utilizing advanced algorithms, AI can identify patterns and trends that may not be apparent to human experts, enabling more accurate predictions of a patient’s prognosis and response to treatment^5^. This has the potential to significantly enhance personalized healthcare and improve overall patient care.

In this umbrella review, we aimed to highlight the most important anatomic scopes of virtual reality and artificial intelligence in guiding pediatric cardiac interventions via analysis of the most important reviews of literature tackling this matter. In addition, we aimed at highlighting the most important AI models employed in the cardiology practice.

## Methodology

### Registration

This umbrella review is registered in PROSPERO, registration ID 1080436.

### Objectives

This umbrella review aims to examine relevant literature reviews to identify the most important anatomical areas where virtual reality (VR) and artificial intelligence (AI) are currently guiding pediatric cardiac interventions. It also identifies the most used AI models in cardiology and assesses the current state of VR and AI integration in cardiac care, highlighting advancements and challenges.

### PICO framework

• **Population (P):** Pediatric and adult patients undergoing cardiac interventions and cardiac surgeries.
• **Intervention (I):** Application of Virtual Reality (VR) and Artificial Intelligence (AI) technologies in cardiac care, encompassing surgical training, procedural planning, risk assessment, and outcome prediction. This includes VR training modules, AI-driven diagnostics, AI-assisted decision-making, and VR-based pre-surgical planning.
• **Comparator (C):** There is no definite comparator group. However, the implicit comparison is to traditional methods of cardiac intervention and surgery *without* the use of VR and AI. This is inferred from the discussion of the benefits and limitations of VR and AI compared to existing practices.
• **Outcome (O):** The outcomes of interest include:
  ∘ Identification of the anatomical scopes of VR and AI application in pediatric cardiac interventions.
  ∘ Identification of the most used AI models in cardiology and assesses the current state of VR and AI integration in cardiac care, highlighting advancements and challenges.

### Search strategy

To ensure comprehensive search, we employed a multi-faceted approach, using a combination of keywords and search strategies to identify relevant literature reviews. The search strategy was built around the core concepts of:

• Virtual Reality and Augmented Reality
• Artificial Intelligence and Machine Learning
• Cardiac Interventions and Surgeries
• Medical Training and Simulation

The following keywords and their combinations were used:

• “reviews”
• “artificial intelligence” OR “machine learning”
• “virtual reality” OR “augmented reality” OR “mixed reality”
• “cardiac interventions” OR “cardiac surgery” OR “heart surgery”
• “pediatric cardiology” OR “congenital heart disease”
• “surgical simulation” OR “medical simulation” OR “surgical training” OR “cardiac training”
• “procedural planning”
• “risk assessment” OR “outcome prediction”

These keywords were combined using Boolean operators (AND, OR) to create specific search strings. Examples include:

• “reviews” AND “artificial intelligence” AND “cardiac surgery” AND “training”
• “reviews” AND “virtual reality” AND “pediatric cardiology” AND “simulation”
• “reviews” AND “machine learning” AND “cardiac interventions” AND “risk assessment”

### Database selection

The following databases were searched from inception to 12 June 2025:

• PubMed
• Scopus
• Web of Science
• Google Scholar

### Inclusion criteria

• Systematic reviews and meta-analyses focused on the application of VR, AR, AI, and machine learning in cardiac interventions, surgeries, training, and planning.
• Reviews specifically addressing the anatomical scopes of these technologies in education, cardiac interventions
• Reviews published in English.

### Exclusion criteria

• Narrative reviews, research articles, editorials, opinion papers, and conference proceedings (to focus on synthesized evidence).
• Non-English articles.
• Grey literature
• Articles not focused on the interplay between VR/AR/AI/ML and cardiac interventions.
• Articles not focused on the anatomical scope of these technologies
• Non-peer-reviewed sources.
• Reviews focusing solely on adult cardiac interventions without relevance to pediatric cardiology.

### Search process

**Figure 1.**
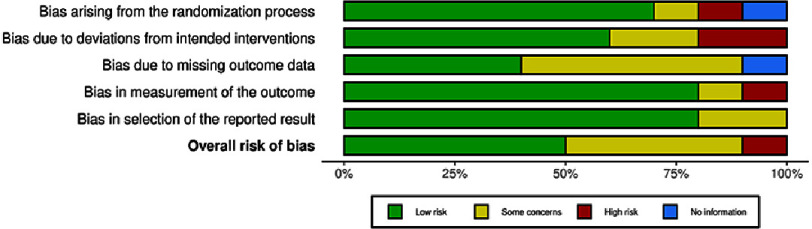
Risk of bias assessment for our umbrella review.

• Initial Search: The initial search was conducted using the keywords and search strings across the selected databases.
• Title and Abstract Screening: All identified records were screened based on their titles and abstracts to assess their relevance to the research question.
• Full-Text Review: Potentially relevant articles were retrieved in full text and assessed against the inclusion and exclusion criteria.
• Reference List Screening: The reference lists of included studies were manually searched to identify additional relevant reviews.
• Data from included reviews were extracted using a standardized data extraction form.
• Data Synthesis: A qualitative synthesis of the findings from included reviews was performed, focusing on the anatomical scopes, AI models, and applications of VR and AI in pediatric cardiac interventions.
• Quality Assessment: The methodological quality was assessed using AMSTAR-2 (Table 1) and Risk of Bias (RoB) was assessed using RoB-2 tool (Figure 1).

### Statistical analysis

Python-based data analysis was conducted to determine the frequency (n) and percentage (%) of each anatomical scope across the following categories: cardiac surgery training, planning of cardiac interventions, and artificial intelligence-driven decision-making in cardiac interventions. Additionally, the specific imaging modalities utilized to facilitate virtual reality (VR) were identified, counted, and expressed as both absolute numbers (n) and percentages (%). Donut charts were employed to visually represent the proportional data where appropriate.

**Table 1:** Summary and heterogeneity of included reviews.

**Feature**	**Arjomandi Rad et al. (2023)**	**Bakhuis et al. (2023)**	**Sulague et al. (2024)**
**Study Design**	Systematic Review	Narrative Review	Systematic Review
**Population/ Intervention**	Simulation-based training in *adult* cardiac surgery. Studies focused on trainees/surgeons of varying experience. Covered wide range of cardiac procedures.	VR simulation and surgical planning in cardiothoracic surgery. Population: Not specifically defined, but implied to be primarily adult (given the focus on general cardiothoracic procedures).	AI applications in cardiac surgery. Studies included various patient populations undergoing cardiac surgery. Most were *adult populations*, but some studies included *pediatric patients.* Diagnoses: Variety of cardiac conditions requiring surgery (e.g., coronary artery disease, valvular heart disease, heart failure, congenital heart defects).
**Key Findings/ Outcomes**	Simulation improves surgical skills, accuracy, and confidence. Limited formal validation of non-tissue models.	Promising tools for VR in cardiothoracic surgery, but large-scale trials are absent as of yet.	AI can improve clinicians’ decisions in cardiac surgery, but more studies are needed to ensure accuracy and safety.
**Methodological Quality**	SR was conducted following Cochrane Collaboration guidelines and PRISMA.	NR was performed using MEDLINE database.	SR was performed from 2000 to 2022 in following databases: PubMed, Embase, Europe PMC, Epistemonikos, CINAHL, Cochrane Central, Google Scholar, Web of Science, Scopus, Cambridge Core, clinicaltrials.gov, and science.
**Strengths**	Comprehensive search strategy.	Provides a broad overview of VR applications and a future outlook.	Good description of the AI Application Characteristics
**Limitations**	Validity assessment is scarce within the field.	Lacks quantitative data. A limited number of published records.	Lacks randomized clinical trials (RCTs) and only included cohort studies. In addition, most of the studies acquired clinical data through database registry.
**Overall Conclusion/ Recommendation**	Simulation provides substantial benefits to trainees; however Further evidence is needed to explore its direct impact on clinical practice.	VR tools will become increasingly integral parts of daily practice in cardiothoracic surgery.	More highly powered studies need to be done to assess challenges and to ensure accuracy and safety for use in clinical practice.

## Results

**Figure 2.**
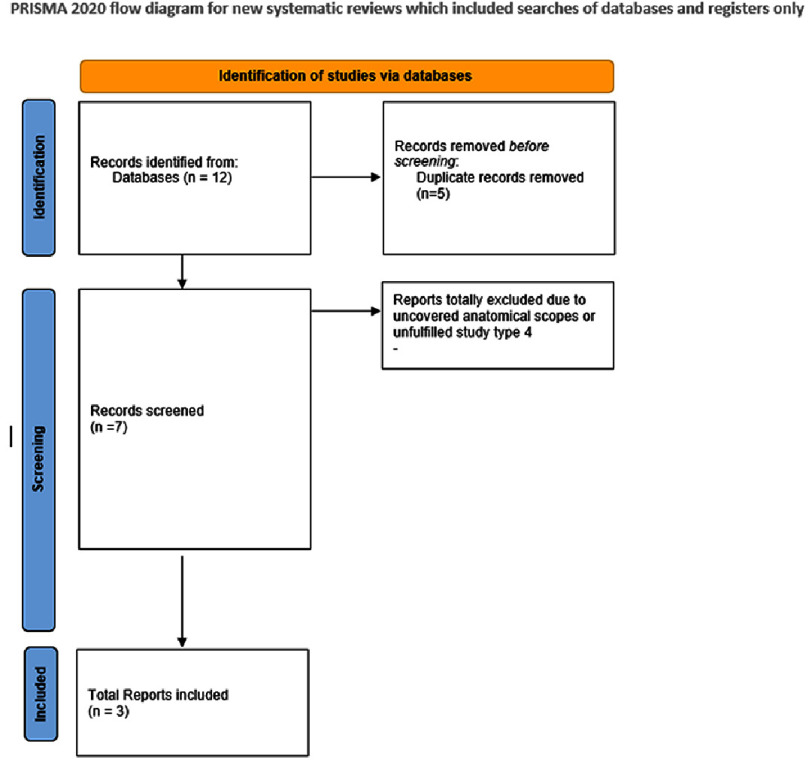
PRISMA flow chart describing the study selection process of this study.

This umbrella review identified three systematic reviews ^6–8^ that meet the inclusion criteria, focusing on the use of augmented/virtual reality in cardiology and/or cardiac surgery for training, pre-procedural planning, and the application of AI in decision-making within the field. Initially, seven studies were considered; however, four were excluded ^9–12^ due to not meeting the study type criteria or lacking coverage of the relevant anatomical scope. (Figure 2 presents the PRISMA flow chart detailing the study selection process, while Table 1 summarizes the main characteristics of the included reviews.)

### Virtual reality in cardiology/cardiac surgery training

The cognitive process of detecting targets in space, recognizing distance and directional relationships, and mentally altering their location is known as spatial skills. The ability to create, preserve, and work with mental pictures in space is known as visual spatial ability (VSA)^1^.

Coming to the medical field, and while it is mandatory to evaluate the spatial ability for admission to undergraduate dental programs and aviation, surgical training programs do not require VSA testing before admission^13^. This might be one of the factors responsible for variations in outcomes of surgeries overall and pediatric cardiac surgeries. An article by Kalun et al. suggested that assessing VSA in surgical trainees is crucial, as findings indicate that individuals with elevated VSA typically show better surgical performance. Additionally, those with higher VSA usually need fewer training sessions to achieve a specific level of performance compared to their counterparts with lower VSA^1^.

The combination of virtual reality and artificial intelligence allows us to overcome the interpersonal variability of VSA. After traditional cross-sectional imaging is completed, a process called segmentation allows specialty engineers to convert 2D DICOM (Digital Imaging and Communications) in Medicine images to 3D VR Maker, which allows trainees and experienced surgeons to interact with those 3D models during pre-surgical planning and can theoretically improve the outcomes of cardiac surgeries and allow medical students with doubts over VSA to choose surgical specialties confidently.

One systematic review was found that discusses the results of VR training in tissue and non-tissue models, in cardiac surgical training^14^. We used this review to determine the number of studies involving immersive virtual reality training in cardiac interventions. We also analyzed the main fields where this technique is being implemented to improve the surgical skills of cardiothoracic residents.

The latter report depicted 27 studies and one catheter-based study was added during search^15–42^; most of these involved training on mitral valve specimens (28%), and coronary surgeries (25%). Other fields of training included mimicking cardiopulmonary bypass, cardiac transplantation, transesophageal echocardiography, and cardiac catheterization.

**Figure 3.**
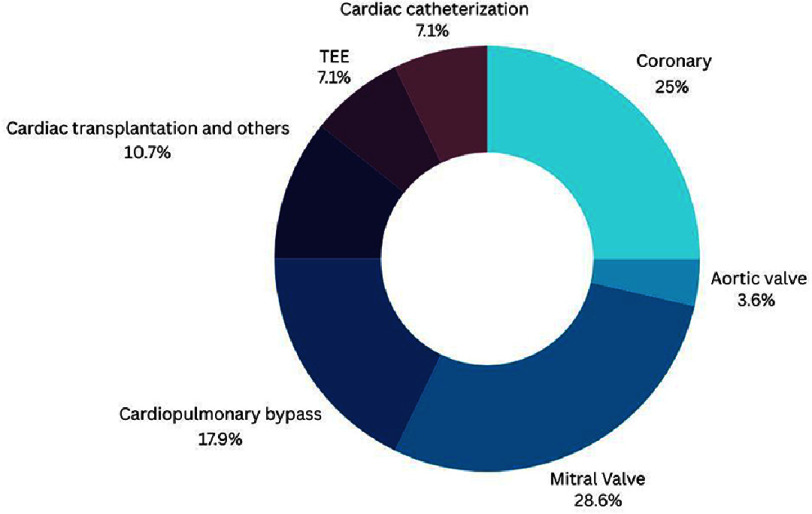
Percentages of scopes of VR-based cardiac surgery training. TEE, transesophageal training.

Figure 3/Table 2 displays the respective percentages of each anatomical field of training.

**Table 2:** Studies involving VR based training in cardiac surgery categorized by the anatomical scope.

	**Non-tissue based**	**Tissue based**
Coronaries (7 studies)	Yasuda^34^ Duffy^31^ Joyce^22^	Sharma^16^ Brandao^15^ Feins^36^ Nesbitt^37^
Aortic valve (1 study)	Russo^33^	
Mitral valve (8 studies)	Sardari^32^ Sardari^30^ Jebran^35^ Premyodhin^26^ Valdis 40 (dry vs. wet)^28^ Valdis 20 (no comparison)^29^	Joyce^38^ Tavlasoglu^39^
Perfusion and cardiopulmonary bypass (5 studies)	Hicks^24^ Fouilloux^25^	Hermsen^23^ Luo^42^ Kenny^19^
Others (3 studies)	Hermsen (Septal myomectomy)^18^	Spooner (Cardiac transplantation)^21^ Zhang (LVAD)^40^
Transesophageal echocardiography (2 studies)	Arango^41^ Smelt^27^	
Cardiac catheter training (2 studies)	Bettati et al., Brown et al.^17,20^	

### Virtual reality in planning cardiac interventions

Current medical practice increasingly utilizes advanced 3D imaging modalities such as magnetic resonance imaging (MRI) and computed tomography (CT) to examine and assess complex congenital heart defects. These modalities enable comprehensive visualization of the cardiovascular anatomy and precise measurements of relevant intracardiac dimensions and volumes^43^. Nevertheless, the conventional representation of 3D anatomy through two-dimensional (2D) slices perpendicular to each other presents challenges in accurately assessing the intricate 3D structures and their spatial relationships. Overcoming these challenges involves reconstructing the patient-specific 3D anatomy using image processing techniques applied to CT or MRI data^44^. While various methods and software tools are available for this reconstruction, manual intervention is often necessary due to the intricate nature of the anatomy and the presence of imaging artifacts. The resulting 3D reconstructions can then be employed to enhance the comprehension of the complex anatomy^45^.

Virtual reality has taken 3D reconstruction to a whole new level where reconstructed models are rendered interactable, with the aid of immersive virtual reality.

Steps include image acquisition and 3D modeling, then several programs are available, to import patient-specific models, and make them compatible with commercially available mounted headsets.

**Figure 4.**
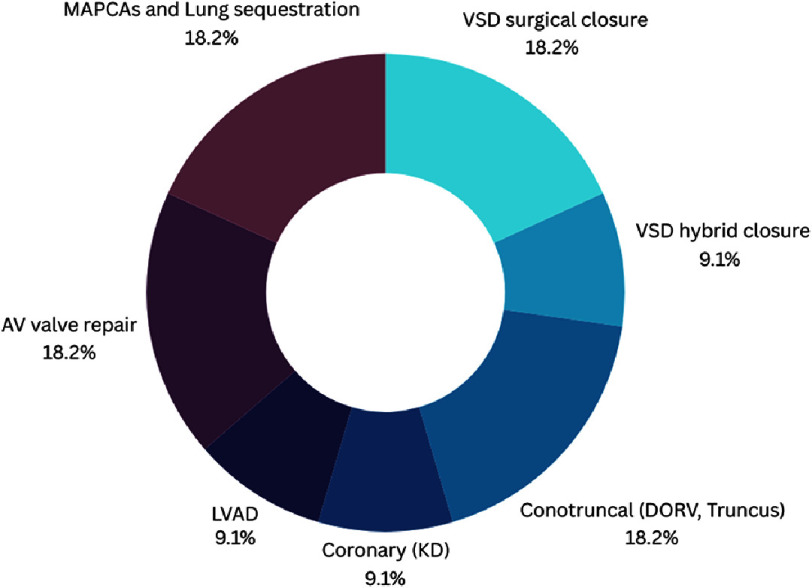
Percentages of distribution of immersive VR assisted cardiac surgeries. AV, Atrioventricular; DORV, Double outlet right ventricle; KD, Kawasaki disease; LVAD, Left ventricular assisted device; MAPCAs, Main aortopulmonary connecting arteries; VSD, Ventricular septal defects.

Our literature search identified a scoping review by Bakhuis et al.^46^, which demonstrated that the number of case studies involving the use of VR in surgical planning for congenital heart disorders is lower than what is seen for education in training, with a total of ten studies, conotruncal anomalies, VSD open surgical closure, Cono-truncal anomalies, AV valve repair and pulmonary sequestration were the main field of the published reports (accounting each for 18%) (Table 3/Figure 4)^47–57^.

**Table 3:** Studies involving immersive VR in pediatric cardiac surgery categorized by the type of treated lesion.

**Field of the study**	**Study**	**Imaging technique used**
VSD surgical closure (2 studies)	Ong et al.^50^ Mendez^51^	CT (computed tomography)
VSD hybrid closure (1 study)	Ghosh^52^	CMR (Cardiac magnetic resonance)
Truncus (1 study)	Ong et al.^50^	CT
MAPCAs (1 study)	Van de Woestijne^56^	CT
DORV (1 study)	Ayerbe^55^	CMR
Atrioventricular valves (1 study)	Pushparajah K^48^	3D Echocardiography
Left ventricular assist device (1 study)	Ramaswamy^53^	CT
Intralobar sequestration Extralobar sequestration Bronchogenic cysts (1 study)	Pelizzo^49^	CT
Coronary revascularization in Kawasaki patient (1 study)	Sadeghi^57^	CT

**Figure 5.**
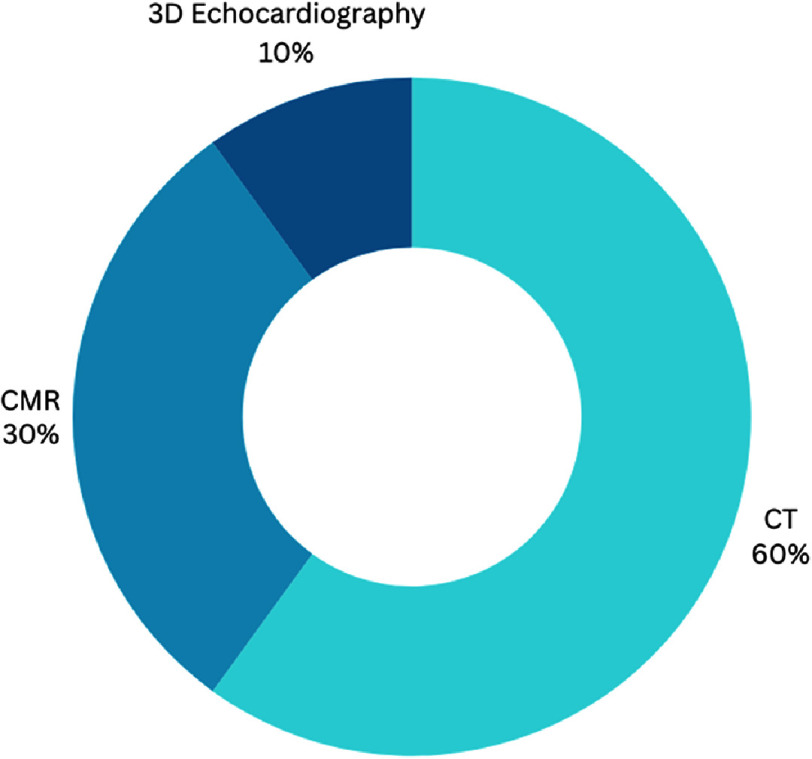
Percentages of distribution of imaging modalities used for VR reconstruction. CMR, Cardiac magnetic resonance imaging; CT, Computed tomography; VR, Virtual reality.

Another aspect worth noting was the imaging modality used for 3D modeling, CT accounted for 60% of the reported studies, while CMR for 30%, and only one case report was performed using 3D echocardiography (Figure 5). This shows the drawback of reconstructive imaging, which relies on either high radiation or lengthy imaging modalities.

There is still a long way to go to develop echocardiographic software, and 3D modalities in this bedside technique to be adaptable to immersive virtual reality and to spare the patients from lengthy, inconvenient procedures and from radiation. Expertise also is another barrier to the generalization of VR reconstructive software^58^.

### Artificial intelligence in cardiac diagnosis and decision making, main models employed and relevant anatomical scopes

The development of advanced deep-learning technology now provides the chance to identify risk indicators that were not previously measurable. This technology can also evaluate intricate, interconnected patterns using easily accessible clinical data for predicting risks. The use of machine learning can serve in diagnosis and decision making. Two main types of machine learning are employed, the feed-forward network which are mainly used for decision making and the convoluted network/filters which serve for image diagnosis.

### Feed forward network and outcome prediction

A feed forward network is a series of interconnected layers, where multiple hidden layers separate the input from the output layer. Machine learning is achieved retrogradely by backward propagation. The data of society of thoracic surgeons and electrocardiogram results are used to feed the input layer and achieve a good accuracy in prediction of cardiac surgery outcome with an area under the curve ranging from 0.85 to 0.9. The main drawback of feed forward network is the need of large volume of input data rendering it non-optimal for processing of images and image diagnosis. Sulague and colleagues^8^, have published recently their preprint which served as a basis for identifying relevant AI-based studies for outcome prediction in cardiac interventions^59,60^.

This review included 33 studies^61–93^, that explored how machine learning can predict certain events such as major bleeding or mortality after different types of cardiac surgeries.

Regarding the specific fields tackled by the predictive model of machine learning, heart transplantation was the most important point of focus, accounting for 38.9% of the studies tackling the role of AI in risk prediction (Figure 6A).

**Figure 6A.**
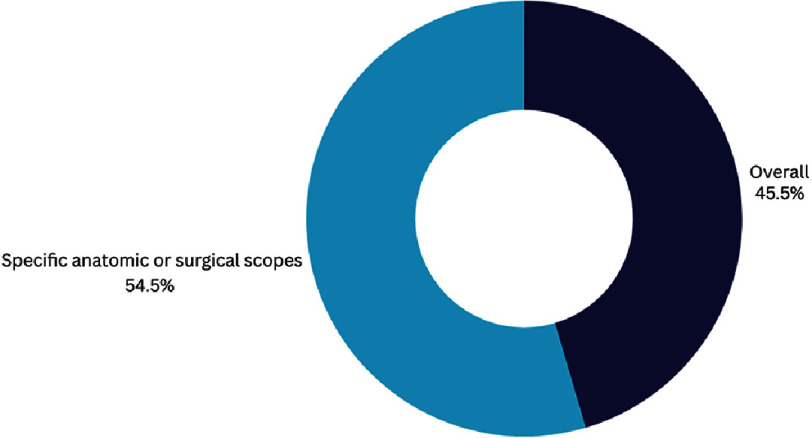
Overview of scopes of AI studies in decision making in cardiac interventions.

**Figure 6B.**
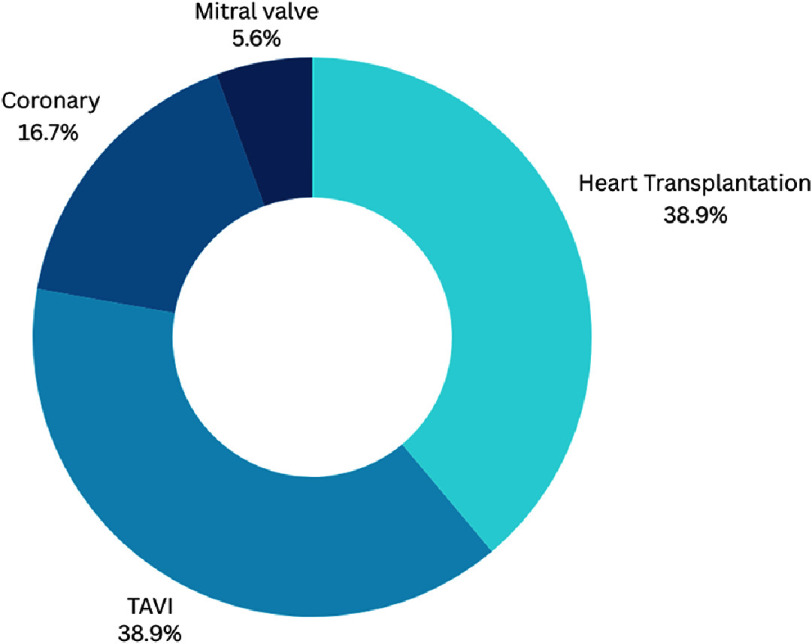
Detailed scopes of AI studies with specific anatomical scope.

**Figure 6C.**
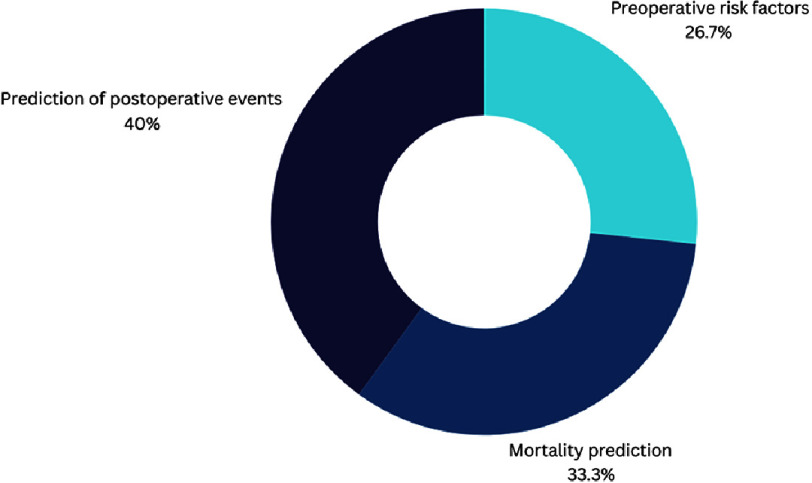
Detailed fields of AI studies with generalized scope.

A new software developed in Sinai Medical Center, aimed at using 12-lead ECG in risk prediction in non-cardiac surgeries. The newly manufactured software, PreOpnet was superior to the routinely used Revised Cardiac score index, in anticipating adverse events after non-cardiac surgeries^87^.

Another recent review, published during the drafting of this manuscript, showed that >672 AI-based devices have already been approved by the FDA and might have an impact on the outcomes of cardiac patients, from planning to risk assessment^94^. (Table 4 and Figures 6A/B/C summarize the main anatomical scopes where AI is helping decision making).

**Table 4:** Studies involving use of AI in decision making regarding cardiac surgery or cardiac catheterization.

Non specified anatomic scope (15 studies)	
	**Decision making:Preoperative and intraoperative risk factors: (4)** Muzio^62^ Bodenhofer^82^ Fernandes^75^ Li^68^**Mortality: (5)** Allyn^81^ Park^70^ Chang^79^ Fan^89^ Molina^92^**Postcardiac events: (6)** Karri^69^ Kim^72^ Aranda^84^ He^87^ Xue^78^ Luo^91^
**Specified Anatomic or surgical scope**	
Coronary (3)	Zea-Vera^80^ Gao^83^ Hu^90^
Heart Transplantation (7)	Kampaktsis^93^ Li^71^ Ayers^88^ Kampaktskis^63^ Shou^67^ Zhou^66^ Agasthi^64^
Transcatheter aortic valve replacement (7)	Hernandez-Suarez^73^ Agasthi^61^ Evertz^86^ Hasimbegovic^65^ Thalappillil^77^ Truong^76^ Kilic^74^
Mitral valve (1)	Jiang^85^

### Convolutional networks and image diagnosis

A convolutional network is a deep-learning neural network employed to analyze grid-like structures by applying a filter (small matrix) to extract required features from a large image and analyze it.

**Figure 7.**
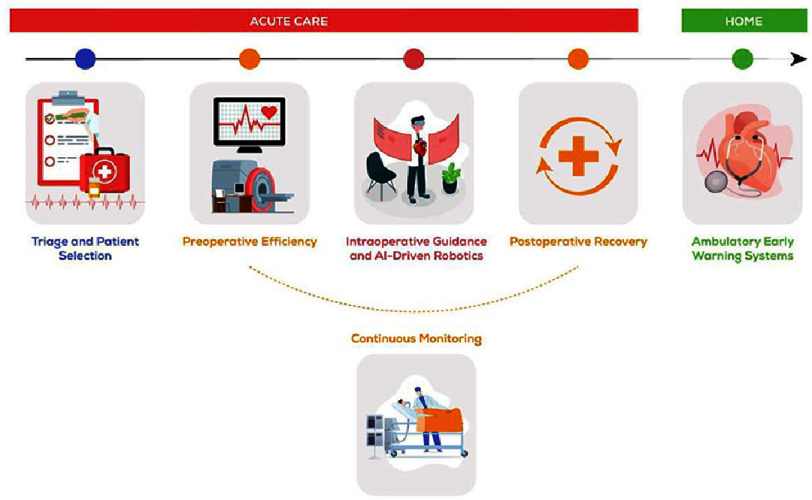
Future uses of AI in cardiac care, a promising journey.

Systems based on convolutional networks can now fully analyze a 12-lead ECG, can help in phase detection in echocardiography and also in localizing important landmarks in cardiac magnetic resonance imaging. (Figure 7 shows the main future uses of artificial intelligence in cardiac care)^95–98^.

## Discussion

This review analyzed the current literature on the applications of VR and AI in cardiac interventions, revealing both promising advancements and significant challenges. The integration of VR in cardiac surgical training demonstrates substantial potential. The findings consistently show that VR training, particularly utilizing immersive techniques, improves surgical skills and reduces the need for extensive training sessions, especially for trainees with lower visual-spatial abilities (VSA)^1,2^. Our analysis of^14^ and subsequent literature uncovered 28 studies focusing on VR training in cardiac interventions, predominantly concentrated on mitral valve procedures (28%) and coronary surgeries (25%)^15–42^. This focus highlights areas where VR’s potential for improved precision and procedural familiarity are most readily apparent. However, the relatively small number of studies in certain areas like cardiac transplantation (10.7%) indicates a significant need for further research to expand the applications of VR training across a wider spectrum of cardiac procedures.

Regarding the use of imaging modalities for 3D modeling in VR, CT scans are predominantly employed (60%), followed by CMR (30%), underscoring the dependence on established imaging techniques (Figure 3). The reliance on CT and CMR presents limitations, such as high radiation exposure or lengthy procedures^58^, and this aspect warrants further investigation into the use of less invasive modalities like 3D echocardiography. Moreover, the need for specialized expertise in both VR and AI technology is a potential barrier to wider implementation and adoption.

Practical examples on how VR can improve accuracy, particularly in pediatric cardiac surgery is highlighted in the individual case series composing the reviews studied in our umbrella review. Ong et al.’s^99^ paper describes the novel use of VR for pre-surgical planning in two infants with complex CHD. In the first case, a 2.95 kg baby girl with truncus arteriosus, VSD, and aortic arch hypoplasia, VR allowed for improved visualization of the VSD and hypoplastic aortic arch, leading to accurate representation of the anatomy. The patient underwent complete repair and made an uneventful recovery, suggesting that VR’s improved visualization aided in successful surgical planning and execution.

In the second case, a 5-month-old infant with a large VSD and right-sided CDH, VR enabled clear visualization of the VSD margins, including relationships to the great vessels, beyond traditional imaging planes. At surgery, the VR model accurately represented surgical findings, including the cardiac shift and rotation. The child tolerated the procedure and weaning from pulmonary hypertensive medications. While the authors stop short of claiming VR *changed* the surgical outcome, they do suggest in both cases, that VR offered improved anatomical understanding preoperatively.

For the truncus arteriosus case, this meant better visualization of the arch; for the VSD/CDH case, improved understanding of the VSD’s margins and the heart’s spatial relationship with surrounding structures. Pushparajah et al. (2021)^97^ focused on atrioventricular (AV) valve repair in 15 pediatric patients and assessed how VR imaging influenced surgical decisions. In 67% of the cases, surgeons reported “more” or “much more” confidence in their understanding of the pathology and surgical approach after reviewing VR images derived from 3D echocardiography. After VR visualization, surgeons would have modified their surgical approach in a majority of cases. The improved clarity of anatomical structures was noted as a main impact. The results show 53% in making minor and 7% major modification.

The use of AI in cardiac diagnosis and decision-making is also highlighted, with two primary models—feed-forward networks for decision-making and convolutional networks for image analysis—emerging as key tools. While studies on the predictive capabilities of AI in cardiac surgery outcome prediction show strong potential with accuracy ranging from 0.85 to 0.9^8^, the limitations of feed-forward networks regarding large data requirements are a crucial factor to address^61–93,100–123^. This limitation is particularly relevant in the context of image-based diagnoses, where convolutional networks might be a more suitable choice. This research also reveals a considerable number of AI-based devices are already approved by the FDA, signifying AI’s growing impact on various aspects of cardiac care^94^. The focus on coronary surgeries and heart transplantation (39% of studies in AI risk prediction) underscores where AI is demonstrating the greatest clinical utility (Figure 5).

The 510(k) clearance process for AI and VR medical devices typically involves demonstrating “substantial equivalence” to a predicate device already legally marketed in the US. First, the company meticulously documents its device’s intended use, technological characteristics, and performance specifications. Then, they identify a suitable predicate device –a similar product already cleared by the FDA. The core of the 510(k) submission lies in proving that the new device is as safe and effective as the predicate. This requires comparative testing, often involving clinical data, to show that the AI or VR device performs at least as well as the predicate in its intended application. For instance, *CathVision*, a company focusing on cardiac electrophysiology, received 510(k) clearance for its *ECGi Lab* system. This system enhances the visualization of intracardiac signals, aiding physicians in identifying and targeting areas for ablation during procedures to treat arrhythmias. Their 510(k) submission would have detailed the ways in which their visualization technology provided equivalent performance to existing cardiac mapping systems, demonstrating that it aided in accurate identification of target areas without compromising patient safety. Another example is *EchoPixel*, which obtained 510(k) clearance for its *True 3D* software. While not exclusively for cardiac applications, True 3D allows physicians to interact with holographic-like 3D renderings of patient anatomy derived from standard CT and MRI scans. In their submission, EchoPixel would have shown that True 3D provides comparable visualization capabilities for pre-operative planning as existing 3D rendering software, enabling surgeons to better understand anatomical structures and plan procedures. Note that specific details of 510k submissions are confidential, so my examples are built on the available information and general regulatory principles.

Implementing AI and VR in cardiac care offers significant cost-effectiveness by improving diagnostic accuracy, enabling early detection of heart conditions, and optimizing treatment plans, which can reduce hospital stays and avoid costly complications. These technologies can streamline workflows, enhance patient engagement, and facilitate personalized medicine, ultimately leading to better outcomes at lower long-term costs. However, initial investment in infrastructure, training, and technology development presents economic challenges, and ensuring the sustainability of AI and VR solutions requires ongoing funding, updates, and integration into existing healthcare systems. Overall, when effectively adopted, AI and VR can contribute to a more efficient, cost-effective, and sustainable approach to cardiac care.

In summary, both VR and AI hold considerable promise for improving cardiac interventions. VR offers realistic training environments, improving skill acquisition and potentially reducing the variability of surgical outcomes associated with VSA differences. AI, with its ability to process vast amounts of data for prediction and analysis, can enhance decision-making and improve the accuracy of diagnoses. However, several challenges must be addressed, including expanding the scope of both technologies across various cardiac procedures, developing less invasive imaging techniques, and addressing the limitations of data-intensive AI algorithms. Future research should focus on refining these technologies, ensuring accessibility and affordability, and systematically evaluating their long-term clinical impact and cost-effectiveness.

### Implementation barriers

Despite the promise of VR and AI, significant barriers hinder their widespread adoption in cardiac care:

• **Technical Complexity:** Implementing and maintaining VR and AI systems require specialized IT infrastructure and expertise, which may be lacking in many healthcare settings.
• **Integration with Existing Workflows:** Seamlessly integrating these technologies into existing clinical workflows and electronic health record (EHR) systems is crucial but can be challenging.
• **Data Availability and Quality:** AI algorithms require large, high-quality datasets for training and validation. Data privacy regulations (e.g., HIPAA) and data silos within healthcare organizations can limit data availability and quality.
• **Lack of Standardization:** The absence of standardized VR and AI platforms and protocols makes it difficult to compare results across different studies and institutions.
• **Physician Acceptance:** Some physicians may be resistant to adopting new technologies, especially if they perceive them as a threat to their autonomy or if they lack confidence in their accuracy and reliability.
• **Patients’ acceptance:** Patients may be resistant to digital transformation in healthcare due to concerns about privacy and data security, particularly with sensitive medical information. There may also be a lack of trust in digital tools compared to traditional in-person interactions. Some patients might find technology intimidating or face difficulties in accessing or using digital platforms due to a lack of digital literacy or resources. Additionally, there may be concerns about the impersonal nature of digital interactions and skepticism about the efficacy and reliability of digital health solutions. Addressing these concerns through education, user-friendly interfaces, and reassuring security measures is crucial to fostering acceptance^124^.

### Ethical implications

Another point worth considering is how those new technologies can trigger ethical dilemmas, particularly AI. Ethical concerns arise regarding informed consent, especially when procedures involve AI-driven decision-making that patients may not fully understand. Additionally, issues of data security and privacy are paramount, as sensitive patient information is used to train AI models, raising questions about data handling and potential misuse. Ensuring equitable access to these advanced technologies is also critical to avoid widening healthcare disparities. Overall, a balance must be struck between leveraging technological innovation to improve care and upholding ethical standards that prioritize patient autonomy, safety, and justice^125,126^.

## Limitations

This umbrella review has several limitations:

• **Small number of included reviews:** The analysis is based on a limited number of systematic reviews and meta-analyses. This restricts the scope of our conclusions and may introduce bias due to the selective reporting of results.
• **Heterogeneity of studies:** The included reviews likely encompass a wide range of study designs, patient populations, and outcome measures. This heterogeneity makes it difficult to draw definitive conclusions about the overall effectiveness of VR and AI in cardiac care.
• **Publication bias:** There is a risk of publication bias, with studies showing positive results being more likely to be published than studies with negative or inconclusive findings. This could overestimate the true benefits of VR and AI.
• **Language bias:** Restricting the search to English-language publications may have excluded relevant studies published in other languages.
• **Search strategy:** Although we made every effort to be comprehensive, it is possible that some relevant publications were missed, as with any literature review.

### Future directions and perspectives

Future research should address the limitations of current literature and focus on:

• **Conducting large-scale, randomized controlled trials:** To rigorously evaluate the clinical effectiveness of VR and AI interventions.
• **Developing standardized VR and AI platforms and protocols:** To facilitate data sharing and comparison of results across different institutions.
• **Investigating the ethical and legal implications of AI in cardiac care:** To address issues such as data privacy, algorithmic bias, and liability.
• **Exploring the use of AI in personalized medicine:** To tailor treatment plans to individual patient characteristics and preferences.
• **Developing less invasive imaging techniques for VR modeling:** To reduce radiation exposure and improve patient comfort.
• While some physicians express concern that AI and VR may eventually reduce the need for their services, a more optimistic view sees **these technologies as enhancing, not replacing, their roles**. VR promises to revolutionize medical and surgical training, improving surgical outcomes through enhanced interaction with 3D models. Similarly, AI’s machine learning capabilities are poised to improve diagnostic accuracy and speed, freeing up healthcare professionals to focus on patient care and professional development. Rather than fearing job displacement, physicians should embrace these technological advancements to augment their skills and improve patient outcomes.

## Declarations

### Ethics approval and consent to participate

Not applicable.

### Consent for publication

Not applicable.

### Availability of data and material

All data are made available within the manuscript.

### Funding

This research received no specific grant from any funding agency, commercial or not-for-profit sectors.

### Authors’ contributions

Conceptualization, AFA, EN; Methodology, AFA, AN, GA, LM, MMA, RA, EN; software, AFA, AN, GA, LM, MMA, RA, EN; investigation, AFA, AN, GA, LM, MMA, RA, EN; resources, AFA, AN, GA, LM, MMA, RA, EN, data curation, AFA, AN, GA, LM, MMA, RA, EN; writing—original draft preparation, AFA, AN, GA, LM, MMA, RA, EN; writing—review and editing, AFA, AN, GA, LM, MMA, RA, EN; supervision, AFA; project administration, AFA; funding acquisition, (non-applicable)

All authors have read and agreed to the published version of the manuscript.

## Competing interests

The authors declare there are no competing interests.

## Acknowledgement

To the peacekeepers in every part of the world, in every community, every family and every tiny relationship. Peace keeping might sometimes look like weakness, but it requires utmost strength. We also wanted to thank Dr. Nadine El Husseiny for providing us with her artwork in the figures of the manuscript.

## References

[1] Sonnadara R, Kalun P, Dunn K, Wagner N, Pulakunta T (2020). Canadian Medical Education Journal Recent evidence on visual-spatial ability in surgical education: A scoping review Des preuves récentes sur les habiletés visuo-spatiales pour la formation en chirurgie: revue exploratoire. Can Med Educ J.

[2] Ntakakis G, Plomariti C, Frantzidis C, Antoniou PE, Bamidis PD, Tsoulfas G (2023). Exploring the use of virtual reality in surgical education. World J Transplant.

[3] Laspro M, Groysman L, Verzella AN, Kimberly LL, Flores RL (2023). The use of virtual reality in surgical training: implications for education, patient safety, and global health equity. Surgeries.

[4] Khor WS, Baker B, Amin K, Chan A, Patel K, Wong J (2016). Augmented and virtual reality in surgery—the digital surgical environment: applications, limitations and legal pitfalls. Annals of Translational Medicine.

[5] Giordano C, Brennan M, Mohamed B, Rashidi P, Modave F, Tighe P (2021). Accessing artificial intelligence for clinical decision-making. Front Digit Heal.

[6] Arjomandi Rad A, Hajzamani D, Sardari Nia P (2023). Simulation-based training in cardiac surgery: a systematic review. Interdiscip Cardiovasc Thorac Surg.

[7] Bakhuis W, Max SA, Maat APWM, Bogers AJJC, Mahtab EAF, Sadeghi AH (2023). Preparing for the future of cardiothoracic surgery with virtual reality simulation and surgical planning: a narrative review. Shanghai Chest.

[8] Sulague RM, Beloy FJ, Medina JR, Mortalla ED, Cartojano TD, Macapagal S, Kpodonu J (2024). Artificial intelligence in cardiac surgery: A systematic review. World Journal of Surgery.

[9] Leivaditis V, Beltsios E, Papatriantafyllou A, Grapatsas K, Mulita F, Kontodimopoulos N, Baikoussis NG, Tchabashvili L, Tasios K, Maroulis I, Dahm M, Koletsis E (2025). Artificial intelligence in cardiac surgery: transforming outcomes and shaping the future. Clin Pract.

[10] Salavitabar A, Dutro M, Zablah JE, Remote Collaboration (2024). Virtual reality for preprocedural planning of complex percutaneous congenital interventions: a case series. J Soc Cardiovasc Angiogr Interv.

[11] Vaidya YP, Shumway SJ (2025). Artificial intelligence: the future of cardiothoracic surgery. Journal of Thoracic and Cardiovascular Surgery.

[12] Farooqi KM, Kalfa D (2021). Commentary: virtual reality in presurgical planning: the future is already here. JTCVS Tech.

[13] Khine MS (2016). Visual-spatial ability in STEM education: transforming research into practice. Vis Abil STEM Educ Transform Res Into Pract.

[14] Arjomandi Rad A, Hajzamani D, Sardari Nia P (2023). Simulation-based training in cardiac surgery: A systematic review. Interdiscip Cardiovasc Thorac Surg.

[15] Brandão CM De A LRP, Dinato FJ, Monteiro R, Fiorelli AI, Jatene FB (2021). Evaluation method of training simulation on biological models for cardiovascular surgery residents. J Card Surg.

[16] Sharma VJ, Barton C, Page S, Ganesh JS, Patel N, Pirone F, Lin Z, Kejriwal NK, El Gamel A, McCormack DJ, Meikle F (2021). Cardiac surgery simulation: a low-cost feasible option in an Australasian setting. ANZ Journal of Surgery.

[17] Brown M, Krishnananthan N, Paul V (2022). Right heart catherisation–a virtual reality. European Heart Journal.

[18] Hermsen JL, Yang R, Burke TM, Dardas T, Jacobs LM, Verrier ED, Mokadam NA (2018). Development of a 3-D printing-based cardiac surgical simulation curriculum to teach septal myectomy. Journal of Thoracic and Cardiovascular Surgery.

[19] Kenny L, Booth K, Freystaetter K, Wood G, Reynolds G, Rathinam S, Moorjani N (2018). Training cardiothoracic surgeons of the future: the UK experience. Journal of Thoracic and Cardiovascular Surgery.

[20] Bettati P, Dormer JD, Young J, Shahedi M, Fei B Virtual reality assisted cardiac catheterization. 82.

[21] Spooner AJ, Faulkner CM, Novick RJ, Kent WDT (2019). Optimizing surgical skills in cardiac surgery residents with cardiac transplant in the high-fidelity porcine model. Innov Technol Tech Cardiothorac Vasc Surg.

[22] Joyce DL, Lahr BD, Maltais S, Said SM, Stulak JM, Nuttall GA, Joyce LD (2018). Integration of simulation components enhances team training in cardiac surgery. Journal of Thoracic and Cardiovascular Surgery.

[23] Hermsen JL, Mohamadipanah H, Yang S, Wise B, Fiedler A, Di Musto P, Pugh C (2021). Multimodal cardiopulmonary bypass skills assessment within a high-fidelity simulation environment. Annals of Thoracic Surgery.

[24] Hicks GL, Gangemi J, Angona RE, Ramphal PS, Feins RH, Fann JI (2011). Cardiopulmonary bypass simulation at the Boot Camp. Journal of Thoracic and Cardiovascular Surgery.

[25] Fouilloux V, Gsell T, Lebel S, Kreitmann B, Berdah S (2014). Assessment of team training in management of adverse acute events occurring during cardiopulmonary bypass procedure: a pilot study based on an animal simulation model (Fouilloux, Team training in cardiac surgery). Perfusion.

[26] Premyodhin N, Mandair D, Ferng AS, Leach TS, Palsma RP, Albanna MZ, Khalpey ZI (2018). 3D printed mitral valve models: affordable simulation for robotic mitral valve repair. Interact Cardiovasc Thorac Surg.

[27] Smelt J, Corredor C, Edsell M, Fletcher N, Jahangiri M, Sharma V (2015). Simulation-based learning of transesophageal echocardiography in cardiothoracic surgical trainees: A prospective, randomized study. Journal of Thoracic and Cardiovascular Surgery.

[28] Valdis M, Chu MWA, Schlachta C, Kiaii B (2016). Evaluation of robotic cardiac surgery simulation training: A randomized controlled trial. Journal of Thoracic and Cardiovascular Surgery.

[29] Valdis M, Chu MWA, Schlachta CM, Kiaii B (2015). Validation of a novel virtual reality training curriculum for robotic cardiac surgery a randomized trial. Innov Technol Tech Cardiothorac Vasc Surg.

[30] Sardari Nia P, Heuts S, Daemen J, Luyten P, Vainer J, Hoorntje J, Cheriex E, Maessen J (2016). Preoperative planning with three-dimensional reconstruction of patient’s anatomy, rapid prototyping and simulation for endoscopic mitral valve repair. Interact Cardiovasc Thorac Surg.

[31] Duffy MC, Ibrahim M, Lachapelle K (2019). Development of a saphenous vein harvest model for simulation-based assessment. Journal of Thoracic and Cardiovascular Surgery.

[32] Sardari Nia P, Daemen JHT, Maessen JG (2019). Development of a high-fidelity minimally invasive mitral valve surgery simulator. Journal of Thoracic and Cardiovascular Surgery.

[33] Russo M, Koenigshofer M, Stoiber M, Werner P, Gross C, Kocher A, Laufer G, Moscato F, Andreas M (2020). Advanced three-dimensionally engineered simulation model for aortic valve and proximal aorta procedures. Interact Cardiovasc Thorac Surg.

[34] Yasuda S, Van den Eynde J, Vandendriessche K, Masuda M, Meyns B, Oosterlinck W (2021). Implementation of a beating heart system for training in off-pump and minimally invasive coronary artery bypass. BMC Surgery.

[35] Saha SJebranA-F, Waezi N, Al-Ahmad A, Niehaus H, Danner BC, Baraki H, Kutschka I (2019). Design and training effects of a physical reality simulator for minimally invasive mitral valve surgery. Interact Cardiovasc Thorac Surg.

[36] Feins RH, Burkhart HM, Conte JV, Coore DN, Fann JI, Hicks GL, Nesbitt JC, Ramphal PS, Schiro SE, Shen KR, Sridhar A, Stewart PW, Walker JD, Mokadam NA (2017). Simulation-based training in cardiac surgery. Annals of Thoracic Surgery.

[37] Nesbitt JC, Julien JSt, Absi TS, Ahmad RM, Grogan EL, Balaguer JM, Lambright ES, Deppen SA, Wu H, Putnam JB (2013). Tissue-based coronary surgery simulation: medical student deliberate practice can achieve equivalency to senior surgery residents. Journal of Thoracic and Cardiovascular Surgery.

[38] Joyce DL, Dhillon TS, Caffarelli AD, Joyce DD, Tsirigotis DN, Burdon TA, Fann JI (2011). Simulation and skills training in mitral valve surgery. Journal of Thoracic and Cardiovascular Surgery.

[39] Tavlasoglu M, Durukan AB, Arslan Z, Kurkluoglu M, Amrahov A, Jahollari A (2013). Evaluation of skill-acquisition process in mitral valve repair techniques: a simulation-based study. J Surg Educ.

[40] Zhang L-F, Feng H-B, Yu Z-G, Jing S, Wan F (2018). Surgical training improves performance in minimally invasive left ventricular assist device implantation without cardiopulmonary bypass. J Surg Educ.

[41] Arango S, Gorbaty B, Tomhave N, Shervheim D, Buyck D, Porter ST, Iaizzo PA, Perry TE (2023). A high-resolution virtual reality-based simulator to enhance perioperative echocardiography training. Journal of Cardiothoracic and Vascular Anesthesia.

[42] Luo X, Luo F, Li B, Li B, Tang Y, Sun H (2020). A tissue-based simulation model for cardiopulmonary bypass cannulation/decannulation training. Perfusion.

[43] Kiraly L (2018). Three-dimensional modelling and three-dimensional printing in pediatric and congenital cardiac surgery. Transl Pediatr.

[44] Yoo SJ, Hussein N, Peel B, Coles J, Arsdell GS va, Honjo O, Haller C, Lam CZ, Seed M, Barron D (2021). 3D modeling and printing in congenital heart surgery: entering the stage of maturation. Front Pediatr.

[45] Batteux C, Haidar MA, Bonnet D (2019). 3D-printed models for surgical planning in complex congenital heart diseases: A systematic review. Front Pediatr.

[46] Bakhuis W, Max SA, Maat APWM, Bogers AJJC, Mahtab EAF, Sadeghi AH (2023). Preparing for the future of cardiothoracic surgery with virtual reality simulation and surgical planning: a narrative review. Shanghai Chest.

[47] Nanchahal S, Arjomandi Rad A, Naruka V, Chacko J, Liu G, Afoke J, Miller G, Malawana J, Punjabi P (2024). Mitral valve surgery assisted by virtual and augmented reality: cardiac surgery at the front of innovation. Perfusion.

[48] Pushparajah K, Chu KYK, Deng S, Wheeler G, Gomez A, Kabir S, Schnabel JA, Simpson JM (2021). Virtual reality three-dimensional echocardiographic imaging for planning surgical atrioventricular valve repair. JTCVS Tech.

[49] Pelizzo G, Costanzo S, Roveri M, Lanfranchi G, Vertemati M, Milani P, Zuccotti G, Cassin S, Panfili S, Rizzetto F, Campari A, Camporesi A, Calcaterra V (2022). Developing virtual reality head mounted display (HMD) set-up for thoracoscopic surgery of complex congenital lung malformations in children. Children.

[50] Ong CS, Krishnan A, Huang CY, Spevak P, Vricella L, Hibino N, Garcia JR, Gaur L (2018). Role of virtual reality in congenital heart disease. Congenit Heart Dis.

[51] Mendez A, Hussain T, Hosseinpour A-R, Valverde I (2019). Virtual reality for preoperative planning in large ventricular septal defects. European Heart Journal.

[52] Ghosh RM, Mascio CE, Rome JJ, Jolley MA, Whitehead KK (2021). Use of virtual reality for hybrid closure of multiple ventricular septal defects. JACC Case Reports.

[53] Ramaswamy R, Marimuthu S, Ramarathnam K, Vijayasekharan S, Rao KS, Balakrishnan K (2021). Virtual reality-guided left ventricular assist device implantation in pediatric patient: valuable presurgical tool. Ann Pediatr Cardiol.

[54] Sadeghi AH, Taverne YJHJ, Bogers AJJC, Mahtab EAF (2020). Immersive virtual reality surgical planning of minimally invasive coronary artery bypass for Kawasaki disease. European Heart Journal.

[55] Ayerbe VMC, Morales MLV, Rojas CJL, Cortés MLA (2020). Visualization of 3D models through virtual reality in the planning of congenital cardiothoracic anomalies correction: an initial experience. World J Pediatr Congenit Hear Surg.

[56] Van de Woestijne PC, Bakhuis W, Sadeghi AH, Peek JJ, Taverne YJHJ, Bogers AJJC (2021). 3D virtual reality imaging of major aortopulmonary collateral arteries: a novel diagnostic modality. World J Pediatr Congenit Hear Surg.

[57] Abjigitova D, Sadeghi AH, Peek JJ, Bekkers JA, Bogers AJJC, Mahtab EAF (2022). Virtual reality in the preoperative planning of adult aortic surgery: a feasibility study. J Cardiovasc Dev Dis.

[58] Bertelli F, Raimondi F, Godard C, Bergonzoni E, Cattapan C, Gastino E, Galliotto F, Boddaert N, El Beheiry M, Masson J-B, Guariento A, Vida VL (2023). Fast-track virtual reality software to facilitate 3-dimensional reconstruction in congenital heart disease. Interdiscip Cardiovasc Thorac Surg.

[59] Karatzia L, Aung N, Aksentijevic D (2022). Artificial intelligence in cardiology: hope for the future and power for the present. Front Cardiovasc Med.

[60] Dabiri Y, Velden AVander, Sack KL, Choy JS, Guccione JM, Kassab GS (2020). Application of feed forward and recurrent neural networks in simulation of left ventricular mechanics. Scientific Reports.

[61] Agasthi P, Ashraf H, Pujari SH, Girardo ME, Tseng A, Mookadam F, Venepally NR, Buras M, Khetarpal BK, Allam M, Eleid MF, Greason KL, Beohar N, Siegel RJ, Sweeney J, Fortuin FD, Holmes DR, Arsanjani R (2021). Artificial intelligence trumps TAVI2-SCORE and corevalve score in predicting 1-year mortality post-transcatheter aortic valve replacement. Cardiovasc Revascularization Med.

[62] Lo Muzio FP, Rozzi G, Rossi S, Luciani GB, Foresti R, Cabassi A, Fassina L, Miragoli M (2021). Artificial intelligence supports decision making during open-chest surgery of rare congenital heart defects. Journal of Clinical Medicine.

[63] Kampaktsis PN, Tzani A, Doulamis IP, Moustakidis S, Drosou A, Diakos N, Drakos SG, Briasoulis A (2021). State-of-the-art machine learning algorithms for the prediction of outcomes after contemporary heart transplantation: results from the UNOS database. Clin Transplant.

[64] Agasthi P, Buras MR, Smith SD, Golafshar MA, Mookadam F, Anand S, Rosenthal JL, Hardaway BW, De Valeria P, Arsanjani R (2020). Machine learning helps predict long-term mortality and graft failure in patients undergoing heart transplant. Gen Thorac Cardiovasc Surg.

[65] Hasimbegovic E, Papp L, Grahovac M, Krajnc D, Poschner T, Hasan W, Andreas M, Gross C, Strouhal A, Delle-Karth G, Grabenwöger M, Adlbrecht C, Mach M (2021). A sneak-peek into the physician’s brain: a retrospective machine learning-driven investigation of decision-making in TAVR versus savr for young high-risk patients with severe symptomatic aortic stenosis. J Pers Med.

[66] Zhou Y, Chen S, Rao Z, Yang D, Liu X, Dong N, Li F (2021). Prediction of 1-year mortality after heart transplantation using machine learning approaches: A single-center study from China. International Journal of Cardiology.

[67] Shou BL, Chatterjee D, Russel JW, Zhou AL, Florissi IS, Lewis T, Verma A, Benharash P, Choi CW (2022). Pre-operative machine learning for heart transplant patients bridged with temporary mechanical circulatory support. J Cardiovasc Dev Dis.

[68] Li Y, Xu J, Wang Y, Zhang Y, Jiang W, Shen B, Ding X (2020). A novel machine learning algorithm, Bayesian networks model, to predict the high-risk patients with cardiac surgery-associated acute kidney injury. Clinical Cardiology.

[69] Karri R, Kawai A, Thong YJ, Ramson DM, Perry LA, Segal R, Smith JA, Penny-Dimri JC (2021). Machine learning outperforms existing clinical scoring tools in the prediction of postoperative atrial fibrillation during intensive care unit admission after cardiac surgery. Hear Lung Circ.

[70] Park J, Bonde PN (2022). Machine learning in cardiac surgery: predicting mortality and readmission. ASAIO J.

[71] Li T, Yang Y, Huang J, Chen R, Wu Y, Li Z, Lin G, Liu H, Wu M (2022). Machine learning to predict post-operative acute kidney injury stage 3 after heart transplantation. BMC Cardiovascular Disorders.

[72] Kim RB, Alge OP, Liu G, Biesterveld BE, Wakam G, Williams AM, Mathis MR, Najarian K, Gryak J (2022). Prediction of postoperative cardiac events in multiple surgical cohorts using a multimodal and integrative decision support system. Scientific Reports.

[73] Hernandez-Suarez DF, Kim Y, Villablanca P, Gupta T, Wiley J, Nieves-Rodriguez BG, Rodriguez-Maldonado J, Feliu Maldonado R, da Luz Sant’Ana I, Sanina C, Cox-Alomar P, Ramakrishna H, Lopez-Candales A, O’Neill WW, Pinto DS, Latib A, Roche-Lima A (2019). Machine learning prediction models for in-hospital mortality after transcatheter aortic valve replacement. JACC Cardiovasc Interv.

[74] Kilic A, Goyal A, Miller JK, Gleason TG, Dubrawksi A (2021). Performance of a machine learning algorithm in predicting outcomes of aortic valve replacement. Annals of Thoracic Surgery.

[75] Fernandes MPB, Armengol de la Hoz M, Rangasamy V, Subramaniam B (2021). Machine learning models with preoperative risk factors and intraoperative hypotension parameters predict mortality after cardiac surgery. Journal of Cardiothoracic and Vascular Anesthesia.

[76] Truong VT, Beyerbach D, Mazur W, Wigle M, Bateman E, Pallerla A, Ngo TNM, Shreenivas S, Tretter JT, Palmer C, Kereiakes DJ, Chung ES (2021). Machine learning method for predicting pacemaker implantation following transcatheter aortic valve replacement. Pacing Clin Electrophysiol.

[77] Thalappillil R, Datta P, Datta S, Zhan Y, Wells S, Mahmood F, Cobey FC (2020). Artificial intelligence for the measurement of the aortic valve annulus. Journal of Cardiothoracic and Vascular Anesthesia.

[78] Xue X, Chen W, Chen X (2022). A novel radiomics-based machine learning framework for prediction of acute kidney injury-related delirium in patients who underwent cardiovascular surgery. Comput Math Methods Med.

[79] Chang Junior J, Binuesa F, Caneo LF, Turquetto ALR, Arita ECTC, Barbosa AC, Fernandes AM da S, Trindade EM, Jatene FB, Dossou P-E, Jatene MB (2020). Improving preoperative risk-of-death prediction in surgery congenital heart defects using artificial intelligence model: A pilot study. PLOS ONE.

[80] Zea-Vera R, Ryan CT, Havelka J, Corr SJ, Nguyen TC, Chatterjee S, Wall MJ, Coselli JS, Rosengart TK, Ghanta RK (2022). Machine learning to predict outcomes and cost by phase of care after coronary artery bypass grafting. Annals of Thoracic Surgery.

[81] Allyn J, Allou N, Augustin P, Philip I, Martinet O, Belghiti M, Provenchere S, Montravers P, Ferdynus C (2017). A comparison of a machine learning model with EuroSCORE II in predicting mortality after elective cardiac surgery: a decision curve analysis. PLOS ONE.

[82] Bodenhofer U, Haslinger-Eisterer B, Minichmayer A, Hermanutz G, Meier J (2021). Machine learning-based risk profile classification of patients undergoing elective heart valve surgery. Eur J Cardio-Thoracic Surg.

[83] Gao Y, Liu X, Wang L, Wang S, Yu Y, Ding Y, Wang J, Ao H (2022). Machine learning algorithms to predict major bleeding after isolated coronary artery bypass grafting. Front Cardiovasc Med.

[84] Aranda-Michel E, Sultan I, Kilic A, Bianco V, Brown JA, Serna-Gallegos D (2022). A machine learning approach to model for end-stage liver disease score in cardiac surgery. J Card Surg.

[85] Jiang H, Liu L, Wang Y, Ji H, Ma X, Wu J, Huang Y, Wang X, Gui R, Zhao Q, Chen B (2021). Machine learning for the prediction of complications in patients after mitral valve surgery. Front Cardiovasc Med.

[86] Evertz R, Lange T, Backhaus SJ, Schulz A, Beuthner BE, Topci R, Toischer K, Puls M, Kowallick JT, Hasenfuß G, Schuster A (2022). Artificial intelligence enabled fully automated cmr function quantification for optimized risk stratification in patients undergoing transcatheter aortic valve replacement. J Interv Cardiol.

[87] He K, Liang W, Liu S, Bian L, Xu Y, Luo C, Li Y, Yue H, Yang C, Wu Z (2022). Long-term single-lead electrocardiogram monitoring to detect new-onset postoperative atrial fibrillation in patients after cardiac surgery. Front Cardiovasc Med.

[88] Ayers B, Sandholm T, Gosev I, Prasad S, Kilic A (2021). Using machine learning to improve survival prediction after heart transplantation. J Card Surg.

[89] Fan Y, Dong J, Wu Y, Shen M, Zhu S, He X, Jiang S, Shao J, Song C (2022). Development of machine learning models for mortality risk prediction after cardiac surgery. Cardiovasc Diagn Ther.

[90] Hu L-H, Betancur J, Sharir T, Einstein AJ, Bokhari S, Fish MB, Ruddy TD, Kaufmann PA, Sinusas AJ, Miller EJ, Bateman TM, Dorbala S, Di Carli M, Germano G, Commandeur F, Liang JX, Otaki Y, Tamarappoo BK, Dey D, Berman DS, Slomka PJ (2020). Machine learning predicts per-vessel early coronary revascularization after fast myocardial perfusion SPECT: results from multicentre REFINE SPECT registry. Eur Hear J - Cardiovasc Imaging.

[91] Luo L, Huang SQ, Liu C, Liu Q, Dong S, Yue Y, Liu KZ, Huang L, Wang SJ, Li HY, Zheng S, Wu ZK (2022). Machine learning–based risk model for predicting early mortality after surgery for infective endocarditis. Journal of the American Heart Association.

[92] Molina RS, Molina-Rodríguez MA, Rincón FM, Maldonado JD (2022). Cardiac operative risk in latin america: a comparison of machine learning models *vs* EuroSCORE-II. Annals of Thoracic Surgery.

[93] Kampaktsis PN, Siouras A, Doulamis IP, Moustakidis S, Emfietzoglou M, Van den Eynde J, Avgerinos DV, Giannakoulas G, Alvarez P, Briasoulis A (2023). Machine learning-based prediction of mortality after heart transplantation in adults with congenital heart disease: A UNOS database analysis. Clin Transplant.

[94] Vaid A, Argulian E, Lerakis S, Beaulieu-Jones BK, Krittanawong C, Klang E, Lampert J, Reddy VY, Narula J, Nadkarni GN, Glicksberg BS (2023). Multi-center retrospective cohort study applying deep learning to electrocardiograms to identify left heart valvular dysfunction. Commun Med.

[95] Zihlmann M, Perekrestenko D, Tschannen M (2017). Convolutional recurrent neural networks for electrocardiogram classification. Comput Cardiol (2010).

[96] Farhad M, Masud MM, Beg A, Ahmad A, Ahmed L, Memon S (2023). Cardiac phase detection in echocardiography using convolutional neural networks. Scientific Reports.

[97] Pushparajah K, Chu KYK, Deng S, Wheeler G, Gomez A, Kabir S, Schnabel JA, Simpson JM (2021). Virtual reality three-dimensional echocardiographic imaging for planning surgical atrioventricular valve repair. JTCVS Tech.

[98] Xue H, Artico J, Fontana M, Moon JC, Davies RH, Kellman P (2021). Landmark detection in cardiac MRI by using a convolutional neural network. Radiol Artif Intell.

[99] Ong CS, Krishnan A, Huang CY, Spevak P, Vricella L, Hibino N, Garcia JR, Gaur L (2018). Role of virtual reality in congenital heart disease. Congenit Heart Dis.

[100] Rigby M (1984). Pediatric cardiology for practitioners. Archives of Disease in Childhood.

[101] Cheung HM, Mok GCF, Lee V, Shing MMK, Li CK (2009). A rare presentation of acute lymphoblastic leukaemia in a teenage girl: heart failure. Hong Kong J Paediatr.

[102] Evans CE, Cober ND, Dai Z, Stewart DJ, Zhao YY (2021). Endothelial cells in the pathogenesis of pulmonary arterial hypertension. European Respiratory Journal.

[103] Kovalchin JP, Forbes TJ, Nihill MR, Geva T (1997). Echocardiographic determinants of clinical course in infants with critical and severe pulmonary valve stenosis. J Am Coll Cardiol.

[104] Javadpour H, Veerasingam D, Wood AE (2002). Calcification of homograft valves in the pulmonary circulation - Is it device or donation related?. Eur J Cardio-Thoracic Surg.

[105] Arab Y, Harahsheh AS, Dahdah N, El-Kholy N, Abed MY, Abu Al-Saoud SY, Agha HM, Alahmadi F, Alamer SR, Ali SAwadhiZAl, Ali MT, Alrabte H, Al-Saloos H, Al-Senaidi KS, Alzyoud R, Awidat N, Bouayed K, Bouaziz A, Boukari R, El Ganzoury MM, Elmarsafawy HM, Elrugige N, Fitouri Z, Kotby A, Ladj MS, Bekkar M, Mouawad P, Salih AF, Suleiman M, Choueiter NF (2024). Kawarabi: administrative Structuring of a multicenter research collaborative to study kawasaki disease in the Arab countries. World J Pediatr Congenit Hear Surg.

[106] Stroeder J, Evans C, Mansell H (2015). Corticosteroid-induced bradycardia: case report and review of the literature. Can Pharm J.

[107] Parks K, Liu X, Reasat T, Khera Z, Baker LX, Chen H, Dawant BM, Saknite I, Tkaczyk ER Metadata of the article that will be visualized in Online. Learning.

[108] Dancami MI Acta Cardiologica Predicting heart failure mortality using the Danish Comorbidity Index for Acute.

[109] Rajda C, Bencsik K, Vécsei LL, Bergquist J (2002). Catecholamine levels in peripheral blood lymphocytes from multiple sclerosis patients. Journal of Neuroimmunology.

[110] AbdelMassih AF, Salem A, Arabi S, Malak L, Marzouk H (2019). Intensity of inflammation as the most important predictor of myocardial involvement in JIA. A 3D echocardiographic study. Acta Reumatol Port.

[111] Ismail NA, Habib SA, Talaat AA, Mostafa NO, Elghoroury EA (2019). The relation between serum Hepcidin, Ferritin, Hepcidin: ferritin ratio, hydroxyurea and splenectomy in children with *β*-thalassemia. Open Access Macedonian Journal of Medical Sciences.

[112] Check PP Paperpal Plagiarism Check.

[113] Hanns P, Paczulla AM, Medinger M, Konantz M, Lengerke C (2019). Stress and catecholamines modulate the bone marrow microenvironment to promote tumorigenesis. Cell Stress.

[114] Tchalova K (2022). A thesis submitted to McGill University in partial fulfillment of the requirements of the degree of Doctor of Philosophy (PhD) ©.

[115] Abdelmassih A, Secondary CA, Author C Abdelmassih A Bulletin of the National Research Centre The heart versus the brain, are they also different when it comes to post-vaccination complications, insights from a systematic review of post-COVID-19 vaccines ADEM.

[116] Rosenthal VD, Jin Z, Memish ZA, Rodrigues C, Myatra SN, Kharbanda M, Valderrama-Beltran SL, Mehta Y, Daboor MA, Todi SK, Aguirre-Avalos G, Guclu E, Gan CS, Jiménez Alvarez LF, Chawla R, Hlinkova S, Arjun R, Agha HM, Zuniga Chavarria MA, Davaadagva N, Mohd Basri MN, Gomez K, Aguilar De Moros D, Tai CW, Sassoe Gonzalez A, Aguilar Moreno LA, Sandhu K, Janc J, Aleman Bocanegra MC, Yildizdas D, Cano Medina YA, Villegas Mota MI, Omar AA, Duszynska W, Belkebir S, El-Kholy AA, Abdulaziz Alkhawaja S, Horhat Florin G, Medeiros EA, Tao L, Tumu N, Elanbya MG, Dongol R, Mioljević V, Raka L, Dueñas L, Carreazo NY, Dendane T, Ikram A, Kanj SS, Petrov MM, Bouziri A, Hung NV, Belskiy V, Elahi N, Bovera MM, Yin R (2023). Multinational prospective cohort study of rates and risk factors for ventilator-associated pneumonia over 24 years in 42 countries of Asia, Africa, Eastern Europe, Latin America, and the Middle East: findings of the International Nosocomial Infection Cont. Antimicrob Steward Healthc Epidemiol.

[117] Rosenthal VD, Yin R, Lu Y, Rodrigues C, Myatra SN, Kharbanda M, Valderrama-Beltran SL, Mehta Y, Daboor MA, Todi SK, Aguirre-Avalos G, Guclu E, Gan CS, Jiménez-Alvarez LF, Chawla R, Hlinkova S, Arjun R, Agha HM, Zuniga-Chavarria MA, Davaadagva N, Basri MNM, Gomez-Nieto K, Aguilar-de Moros D, Tai CW, Sassoe-Gonzalez A, Aguilar-Moreno LA, Sandhu K, Janc J, Aleman-Bocanegra MC, Yildizdas D, Cano-Medina YA, Villegas-Mota MI, Omar AA, Duszynska W, BelKebir S, El-Kholy AA, Alkhawaja SA, Florin GH, Medeiros EA, Tao L, Memish ZA, Jin Z (2023). The impact of healthcare-associated infections on mortality in ICU: a prospective study in Asia, Africa, Eastern Europe, Latin America, and the Middle East. Am J Infect Control.

[118] Seibold JM, Ross A (2023). Subsecond release of catecholamines from CD4+ T lymphocytes. Journal of Immunology.

[119] Flierl MA, Rittirsch D, Huber-Lang M, Sarma JVidya, Award P (2008). Catecholamines - Crafty weapons in the inflammatory arsenal of immune/inflammatory cells or opening Pandora’s box§?. Missouri Medicine.

[120] Auerbach SR, Richmond ME, Lamour JM, Blume ED, Addonizio LJ, Shaddy RE, Mahony L, Pahl E, Hsu DT (2010). BNP levels predict outcome in pediatric heart failure patients post hoc analysis of the Pediatric Carvedilol Trial. Circ Hear Fail.

[121] Abdelmassih A, Secondary CA Author C Bulletin of the National Research Centre The heart *vs.* brain, are they also different when it comes to post vaccination complications, insights from a systematic review of post-COVID-19 vaccines ADEM.

[122] Bertrand É, Caru M, Harvey A, Dodin P, Jacquemet V, Curnier D (2023). Cardiac electrical abnormalities in childhood acute lymphoblastic leukemia survivors: a systematic review. Cardio-Oncology.

[123] Paper OS Acta Cardiologica Prevalence of sarcopenia in heart failure with mildly reduced ejection fraction and its impact on clinical outcomes.

[124] Inampudi S, Rajkumar E, Gopi A, Vany Mol KS, Sruthi KS (2024). Barriers to implementation of digital transformation in the Indian health sector: a systematic review. Humanit Soc Sci Commun.

[125] Harishbhai Tilala M, Kumar Chenchala P, Choppadandi A, Kaur J, Naguri S, Saoji R, Devaguptapu B (2024). Ethical considerations in the use of artificial intelligence and machine learning in health care: a comprehensive review. Cureus.

[126] Strika Z, Petkovic K, Likic R, Batenburg R (2024). Bridging healthcare gaps: a scoping review on the role of artificial intelligence, deep learning, and large language models in alleviating problems in medical deserts. Postgraduate Medical Journal.

